# Special Issue “Recent Advances in Neonatal Sepsis”

**DOI:** 10.3390/jcm12041385

**Published:** 2023-02-09

**Authors:** Kosmas Sarafidis

**Affiliations:** 1st Department of Neonatology, School of Medicine, Aristotle University of Thessaloniki, 49 Kostantinoupoleos Str., 54642 Thessaloniki, Greece; kosaraf@auth.gr; Tel.: +30-2310892426

Perinatal medicine and neonatology have seen significant advancements in recent decades. Nevertheless, sepsis remains a major medical problem in neonates [1]. Depending on the time of clinical presentation and the origin of the causative pathogens, it is defined as early-onset sepsis (EOS) when occurring ≤72 h after birth (or by others during the first 7 post-natal days), and as late-onset sepsis (LOS) when it presents thereafter [2,3]. Necrotizing enterocolitis (NEC) is another potentially hazardous bowel disease, primarily affecting premature infants, which is characterized—similarly to sepsis—by inflammation [4]. Moreover, both conditions present with non-specific and often overlapping clinical signs and symptoms, rendering the differential diagnosis difficult. This fact may partly explain the high morbidity and mortality of neonatal sepsis and NEC, especially in preterm infants [1,5,6,7]. Thus, the ongoing scientific effort to better understand the pathophysiological mechanisms of neonatal sepsis and NEC and, ultimately, to improve early diagnosis and treatment should not come as a surprise.

The aim of this Special Issue of the *Journal of Clinical Medicine* referring to the research topic “Recent Advances in Neonatal Sepsis” includes a more in-depth understanding of sepsis (and NEC) pathophysiology as well as the development of diagnostic–prognostic biomarkers or of novel adjunctive or preventive interventions. Herein, an overview of four original articles and one review article included in this Special Issue is provided.

Several biomarkers have been used for the evaluation of neonatal sepsis and NEC, which can be categorized into (a) hematological indices (white blood cell, absolute neutrophil and platelet counts), (b) acute-phase reactants such as C-reactive protein (CRP) and (c) immunological markers (cytokines, chemokines, adhesion molecules, etc.) [8,9,10]. It is disappointing, though, that despite years of research, no biomarker has been shown to reduce morbidity or mortality in neonatal sepsis and NEC, probably due to their relatively low diagnostic accuracy.

In a retrospective study, Cao et al. investigated the relationship of various demographic and clinical factors related to pregnancy and neonates with EOS or LOS. In line with the existing knowledge [3], an association of common perinatal factors and neonatal conditions (birth gestational age, gestational age, chorioamnionitis, persistent pulmonary hypertension, respiratory distress syndrome) with the risk of EOS was documented. Moreover, the increased difference between central and peripheral temperature was significantly associated with LOS. In parallel, the study provided further evidence as to the diagnostic role of circulating interleukin-6 (IL-6) and CRP in neonatal sepsis. IL-6 was found to be a reliable early indicator of both EOS and LOS, and a cut-off value of 100 ng/L was proposed. A CRP value above 5.5 mg/L at the time of sepsis workup was reported to predict culture-positive EOS with a sensitivity of 80% and a specificity of 74%. Of note, no cut-off value could be determined for CRP in culture-positive LOS, which was attributed to the slow elevation in the specific acute-phase protein. As emphasized by the authors, the low sensitivity of blood cultures hinders the diagnostic accuracy of IL-6 and CRP. Blood culture positivity in neonates (still considered the gold standard in neonatal sepsis) is approximately 50–60% [6,11]. Additionally, bilirubin and lactate used in adult sepsis scores [12] were not found to be of value in neonatal sepsis.

The final objective of the study was to evaluate whether throat and rectal swabs obtained from neonates receiving intensive care, weekly after birth, could predict sepsis-causing organisms. The results showed that neonatal colonization and infection merely developed in a parallel fashion, which made the evaluation of microbial colonization less predictive. Therefore, microbial screening is probably more useful for the surveillance of multidrug-resistant microbes and isolation of colonized infants than for predicting neonatal sepsis or identifying the microbiological cause of sepsis.

Immature to total neutrophil granulocytes ratio (I/T ratio) is a laboratory criterion commonly used in neonatal sepsis [13]. In a retrospective study, Wettin et al. compared two ways of measuring white blood cell differentials (and I/T ratio determination): traditional (manual using a microscope) versus automated using flow cytometry. The latter is less time-consuming, while the machine-calculated I/T ratio is supposed to be more accurate, as the number of measured cells is much larger (manually, only 100 randomly selected cells are evaluated per blood smear).

Immature granulocyte counts and I/T ratios with both approaches were assessed in 270 preterm and term infants who were grouped as unlikely or likely to have an infection (based on the interleukin-6 or CRP levels at the time of baseline examination and up to 48 h later, and the results of blood cultures). With automated measurement, infants with probable infection as well as those with a positive blood culture had significantly lower numbers of immature granulocytes and I/T ratios compared to those without infection. This contrasts with manual cell differentiation, where an increase in the I/T ratio was found as expected with infection. The classification of cell bands as mature neutrophil granulocytes by the automated machine explains this inconsistency. Nevertheless, this is something that neonatologists should be aware of, as a low I/T ratio, when determined by the specific hematology analyzer, should not be reassuring but, on the contrary, raise suspicion of an emerging serious infection. It is worth noting that with both techniques, the predictive accuracy of the immature granulocyte count and I/T ratio was poor for predicting infection in neonates.

Metabolomics is a new analytical method that detects metabolites of an organism in a biological sample. These metabolites reflect the state of the organism in both normal and pathological conditions and can be linked to specific biochemical pathways [10]. At present, there is a growing preference for metabolomics as a research tool to investigate the pathophysiology of various diseases and to discover diagnostic/prognostic biomarkers. Thus, the use of multi-omics (including metabolomics) and machine learning technologies and approaches is truly a “golden opportunity” to improve the early diagnosis of neonatal sepsis and NEC [10,14], bringing precision medicine even closer [15].

In this context, Thomaidou et al. conducted untargeted metabolomic analysis using liquid chromatography-mass spectrometry to explore metabolic changes related to LOS and NEC. In this prospective case–control study, a number of metabolites possibly identified as phosphatidylcholines or lysophosphatidylcholines were found to be significantly reduced both in neonates with LOS and those with NEC compared to controls. Additionally, increased L-carnitine could efficiently discriminate neonates with NEC. The results of the study suggested that certain phospholipids and their derivatives could possibly be used as biomarkers for the early detection of LOS and NEC.

This study adds new information to the limited data regarding serum/plasma metabolomics in neonates with LOS or NEC. In fact, it is the first study to evaluate changes in serum phospholipids in neonatal inflammatory conditions such as LOS and NEC. Of note, despite the invasiveness of blood compared to urine sampling, and the associated risk of iatrogenic anemia, especially in preterm infants, blood serum was used for the metabolomic analysis. It was expected to better reflect pathophysiological and metabolic changes in systematic illnesses, such as sepsis and NEC. On the other hand, the small size of the study did not allow the evaluation of the impact on the metabolic profiles of other important variables, such as the type of microbes, cases of confirmed versus possible sepsis/NEC, feeding mode and mortality.

Undoubtedly, other studies are needed not only to verify these findings but also to expand the knowledge on the role of lipids in inflammation. Existing data in adults indicate a disturbed lipid homeostasis in sepsis [16]. Similarly, the value of phospholipids as diagnostic–prognostic biomarkers in neonates with sepsis or NEC remains to be explored with the development of prospective studies. Preferably, metabolomics and gut microbiota analysis should be investigated in parallel, especially when the gut is a major contributor to the disease pathophysiology, like in NEC [17]. All these scientific questions could be the motivation for the development of larger studies in the future.

Antibiotics play a crucial role in the treatment of neonatal sepsis, which largely remains supportive. To counteract the deficiencies of innate and acquired immunity, various immunomodulatory agents (immunoglobulin, granulocyte colony-stimulating factor, etc.) have been attempted as adjuvant treatments in septic neonates. None of them, however, was found to confidently reduce sepsis-related mortality [18]. On the other hand, vaccination or limited exposure to non-infectious bacterial components was reported to augment the neonatal immune response, generating a protective effect against other pathogens. In a study conducted in Africa (Guinea-Bissau), early BCG vaccination was associated with a 43% reduction in infectious deaths within 28 days after birth among low-weight infants [19]. Additionally, in the very low birth weight infants, not only did EOS not increase the risk of LOS, but, surprisingly, it also appeared to be reduced in the smallest, most premature neonates [20].

In light of the so-called “trained immunity” [21], Nakasone et al. investigated whether intraperitoneal administration of a low non-lethal dose of septic challenge, when given three days prior to lethal sepsis induction, could improve mortality in mice. Beyond the significantly higher survival, several inflammatory genes and lipid mediators were significantly depressed in the liver of the pre-treated animals 6 h after the induction of lethal sepsis. These encouraging findings give hope for the development of novel immunomodulating treatments to help reduce mortality, especially in preterm infants with LOS. One should bear in mind, though, that immunomodulation is not without the theoretical risk of unwanted side effects.

Host defense against early-life infections including chorioamnionitis, neonatal sepsis or NEC relies primarily on innate immunity, in which antimicrobial peptides (AMPs) play a major role. AMPs are small peptides naturally produced by various living organisms, although they may be synthetically produced after modification of natural AMPs [22].

In an in-depth review, Agakidou et al. provide the most recent scientific knowledge on human AMPs, describing their antimicrobial–immunomodulating properties, their production sources and their changes in association with pre- and post-natal infections and NEC. Moreover, the potential utility of AMPs in early-life infections as diagnostic or predictive tools is discussed. Considering the increasing antibiotic resistance in neonatal intensive care units [23], perhaps, the most fascinating challenge is the possible use of AMPs, therapeutically or as an adjunctive treatment to antibiotics.

As the Guest Editor, I would like to thank all the authors for their valuable contributions, the reviewers for their time in critically reviewing the papers and making important comments and the *JCM* editorial team for their collective support and assistance.
